# Regeneration of the Fibula with Unidirectional Porous Hydroxyapatite

**DOI:** 10.1155/2019/9024643

**Published:** 2019-10-13

**Authors:** Koji Demiya, Toshiyuki Kunisada, Eiji Nakata, Joe Hasei, Toshifumi Ozaki

**Affiliations:** ^1^Department of Orthopaedic Surgery, Okayama University Graduate School of Medicine, Dentistry and Pharmaceutical Sciences, Japan; ^2^Department of Medical Materials for Musculoskeletal Reconstruction, Okayama University Graduate School of Medicine, Dentistry and Pharmaceutical Sciences, Japan

## Abstract

A fibula graft is one of the most common orthopedic procedures for reconstruction of a bone defect, and some complications related to persistent defects of the fibula have been reported previously. We believe that regeneration of the fibula may be critical for postoperative function and prevention of complications. This report describes a 9-year-old female with Ewing sarcoma of the pelvis who was treated with the double-barrel fibula grafts for pelvic bone defect following tumor resection. The defect after fibular resection was filled with unidirectional porous hydroxyapatite (UDPHAp) implants. A plain radiograph revealed new bone formation and a callus-like structure at one month after surgery and bony union between each UDPHAp implant 5 months after surgery. Resorption of implanted UDPHAp was identified, and partial remodeling of the bone marrow cavity could be seen 1 year 2 months after surgery. A radiograph at final follow-up (5 years 10 months after surgery) demonstrated almost complete absorption of the implanted UDPHAp and clear formation of the cortex and bone marrow in the resected part of the fibula. The patient is able to walk well without any walking supports and to take part in sports activities.

## 1. Introduction

A fibula graft is a standard technique used to reconstruct bone defects in reconstructive orthopedic surgery. Although it remains uncertain whether a segmental fibular defect should be restored, some complications related to persistent defects of the fibula have been reported previously [1, 2]. We believe that regeneration of the fibula may be critical for postoperative function and prevention of complications, especially for juvenile and adolescent patients. We implanted unidirectional porous hydroxyapatite (UDPHAp, REGENOS®, Kuraray Co., Ltd., Tokyo, Japan) into a segmental defect after harvesting a fibula graft in a 9-year-old patient and assessed the radiological and clinical outcome. To our knowledge, there has been no English-language report regarding clinical and radiological assessment of hydroxyapatite implantation into a fibula defect. We therefore report a case of restoration of bone marrow cavity in the fibula defect after implantation of hydroxyapatite, with good regeneration.

## 2. Case Presentation

**Figure 1:**
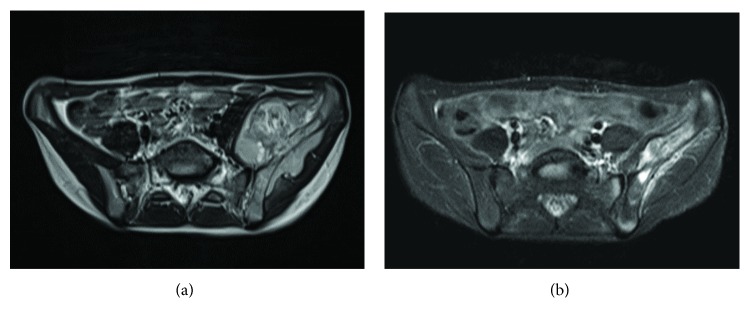
T2-weighted MRI showing a huge extraskeletal mass of pelvic Ewing sarcoma at diagnosis (a) and good response after preoperative chemotherapy (b).

A 9-year-old girl was diagnosed with Ewing sarcoma of the left ilium. Magnetic resonance imaging (MRI) showed an extraosseous bulging mass (Figure 1(a)). She was treated with chemotherapy according to the EURO-E.W.I.N.G.99 (EUROpean Ewing Tumour Working Initiative of National Groups; Ewing Tumour Studies 1999) protocol, followed by radiation therapy. MRI after chemotherapy resulted in remarkable tumor reduction (Figure 1(b)). The patient then underwent surgical resection with an adequate wide margin including the ilium, the sacroiliac joint, and the partial sacrum, and the acetabulum was preserved. A free fibular graft was obtained from the left leg, and the double-barrel fibula grafts were implanted between the osteotomy sites of the iliac bone above the acetabulum and the sacrum. The fibular grafts were fixed with screws, and temporary external fixation was applied to stabilize the reconstruction. She completed postoperative chemotherapy according to the protocol and was followed up regularly at our outpatient clinic.

**Figure 2:**
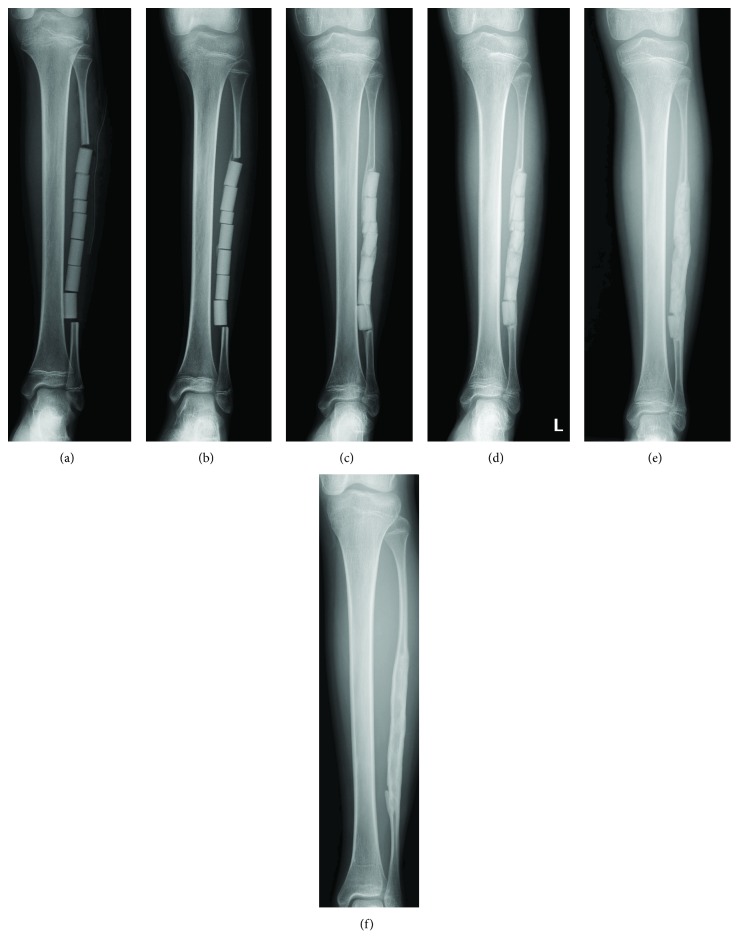
Plain radiographs showing serial changes of implanted UDPHAp, just after surgery (a), at 1 month after surgery (b), 9 months (c), 1 year 2 months (d), 2 years 1 month (e), and 5 years 10 months (f).

To obtain the fibular graft, we dissected along the anterior surface of the septum between the peroneus longus and the soleus muscle. The fibula was exposed by retracting the peroneal muscles anteriorly and incising the periosteum. Subperiosteal detachment was performed cautiously on each of the fibula surfaces. Then, osteotomy was performed proximally and distally. The length of the harvested fibula was 14 cm. The defect after fibular resection was filled with six 2 cm-long column-shaped UDPHAp implants and one 1 cm-long column-shaped UDPHAp implants (Figure 2(a)). The remaining periosteum was sutured to cover the implanted UDPHAp as completely as possible. No cast or brace was applied postoperatively for the lower leg. Weight-bearing on the left leg was not allowed within 6 weeks after surgery in order to stabilize the pelvic reconstruction.

A plain radiograph at one month after surgery revealed new bone formation in the gap between the remaining proximal fibula and the implanted UDPHAp, and a callus-like structure around the centrally implanted UDPHAp (Figure 2(b)). Bony union between each UDPHAp implant was observed 5 months after surgery, and slight proximal migration of the remaining distal fibula was identified without ankle valgus deformity (Figure 2(c)). Resorption of implanted UDPHAp was identified, and partial remodeling of the bone marrow cavity could be seen 1 year 2 months after surgery (Figure 2(d)). Absorption of implanted UDPHAp and new bone formation had progressed 2 years 1 month after surgery, and a bone marrow cavity was partially formed in the implanted UDPHAp (Figure 2(e)). A radiograph at final follow-up (5 years 10 months after surgery) demonstrated almost complete absorption of the implanted UDPHAp and clear formation of the cortex and bone marrow in the resected part of the fibula (Figure 2(f)).

**Figure 3:**
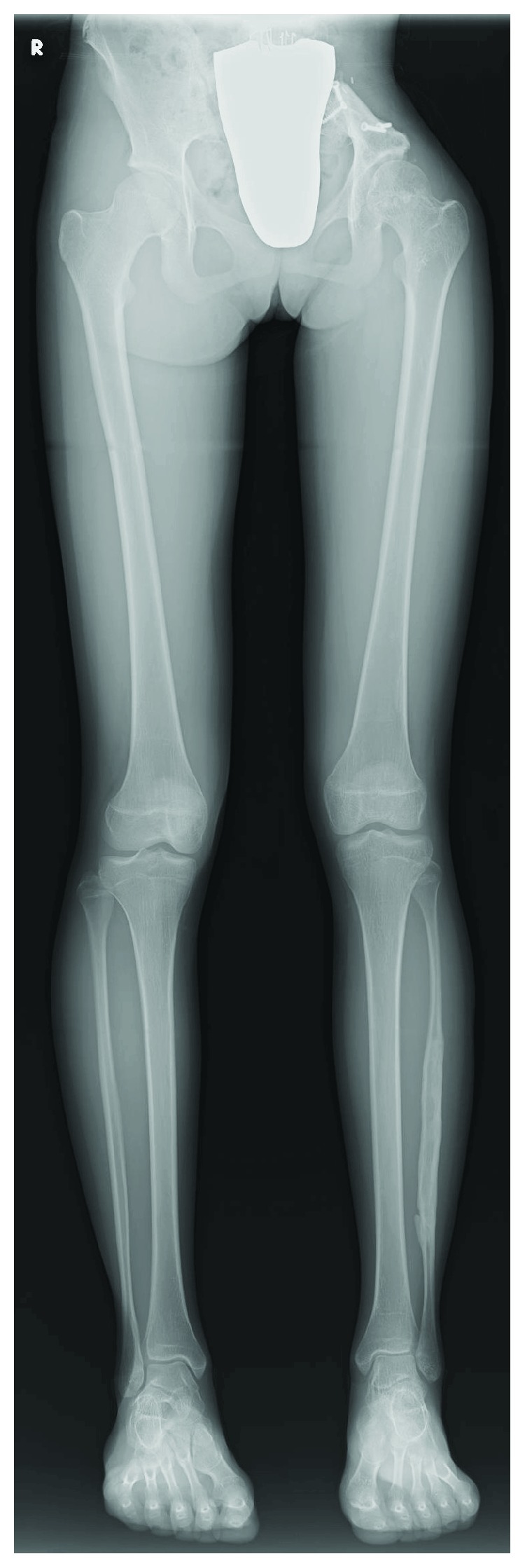
Plain radiograph of the patient's whole legs in a standing position at 5 years 10 months showing regeneration of the left fibula.

At present, the patient is able to walk well without any walking supports and to take part in sports activities. Squatting and Japanese sitting are also possible. There were no surgical complications related to bone harvesting or implantation of UDPHAp. The length between the femoral head and the medial malleolus of tibia is equal on both sides, although there is a 1.5 cm limb length discrepancy due to tumor resection and pelvic reconstruction (Figure 3). The patient has been continuously disease-free with no recurrence of the tumor, and her Musculoskeletal Tumor Society score was 29/30 points (97%) 5 years 10 months after surgery.

## 3. Discussion

Hydroxyapatite (HA) has been extensively used as a bone graft substitute for bone defects in orthopedic surgery since it has sufficient strength, osteoconductive ability, and similarity to the mineral component of the bone. However, conventional HA (first generation) implants have shown poor new bone ingrowth into the pores of the materials due to closed structures with few interpore connections, and newly formed bone with HA implants at defect sites can be fragile and sometimes prone to fracture [3, 4]. Tamai et al. reported good clinical results of novel hydroxyapatite ceramics with an interconnected porous structure, which can improve these downsides of conventional HA and lead to good new bone ingrowth into the graft material [4]. Unidirectional porous hydroxyapatite (UDPHAp) has 75% porosity and 99.9% purity with an interconnected porous structure, and its most distinctive feature is that it consists of unidirectional oval pores oriented toward the horizontal direction that completely penetrate through the material [5]. The pore size (approximately 100-300 *μ*m in the longest diameter) and microstructure can facilitate the invasion of cells and fluids necessary for osteogenesis [6, 7]. Good clinical outcomes of UDPHAp implantation into various bone defects have been reported previously [8, 9].

A fibula graft is one of the most common orthopedic procedures for reconstruction of a bone defect, such as after resection of a bone tumor. Reconstruction of the fibula defect after harvesting might not be necessary, because the fibula and interosseous membrane carry only 6% to 16% of the load applied to the lower extremity [10, 11] and spontaneous regeneration of the fibula has been reported previously, especially in children [12]. However, incomplete regeneration or nonunion following fibula harvesting has also been described [1, 13], and the loss of the fibula after its removal sometimes results in significant donor site morbidity, such as surgical scar pain, weaknesses of plantar flexion of the ankle [14], valgus deformity of the ankle [1], ankle instability [15], and tibial fracture [2]. Our case demonstrated slight proximal migration of the remaining distal fibula without ankle valgus deformity, which could be avoided by using temporary syndesmotic screw fixation. To our knowledge, there is only one comparative study (in Japanese) demonstrating that there were few complications related to harvesting of the fibula in patients with HA implantation into the donor site compared to those without HA [16]. We considered that regeneration of the fibula in the defect could be critical to minimize these morbidities following harvesting of fibula grafts, especially for pediatric patients, so we implanted the UDPHAp into the fibula defect in the current patient.

There are few papers regarding the use of HA as a bone filling material at the fibula donor site. Fujibayashi et al. used conventional HA (first generation) as a spacer to fill the defect in the fibula and reported that in 88% of the patients, newly formed bone was observed around the HA spacer [16]. However, 25% of the patients showed breakage of the HA spacer and/or the wire stabilizing the spacer to the remaining fibula, indicating that bone ingrowth into the pores of the conventional HA (first generation) might be insufficient. In the present case, new bone formation was observed around and inside the implanted UDPHAp only one month after surgery and there was good regeneration of the fibula with the bone cortex and marrow 2 years after surgery. Moreover, the bony continuity of the fibula was complete in the segmental defect after harvesting the fibula. We believe that there are several reasons for this finding: first, the structural features of UDPHAp can lead to good bone regeneration. As discussed above, unidirectional pores in the horizontal direction with some interpore connections can facilitate cellular and blood migration and invasion into the material, which is beneficial for osteogenesis. Second, this is a pediatric patient. Arai et al. assessed the outcome of beta-tricalcium phosphate (B-TCP) implantation into a fibula defect after harvesting [17]. B-TCP is also a useful material to fill bone defects and has been used as a bone filling material for the fibula donor site. They showed that B-TCP was replaced by newly formed bone at an average of 3.2 months after surgery in all children, but bony continuity between the regenerated fibula and the distal fibular end was relatively uncommon in adults. Third, we preserved the periosteum when harvesting the fibula, since the intact periosteum could be one of the important contributors to good bone formation [18].

The implantation of UDPHAp into the segmental defect of the fibula resulted in rapid bone formation around the material and good regeneration of the fibula. There was no complication related to harvesting the fibula or to the implantation of UDPHAp. The trabecular bone was successfully reconstructed at the time of final evaluation (5 years 10 months after surgery). UDPHAp can therefore be a good bone substitute to fill the segmental defect left in the fibula by harvesting the graft.
